# Efficacy and safety of Lianhua Qingwen granule in the treatment of non-influenza viral pneumonia: a randomized, double-blind, placebo-controlled, multicenter clinical study

**DOI:** 10.3389/fmed.2023.1302219

**Published:** 2024-01-17

**Authors:** Chengjie Ma, Bojun Chen, Yanming Li, Li Gu, Jianping Dong, Zhenyang Xu, Lijuan Wei, Zhihong He, Xiuhong Nie, Shuwen Feng, Bin Cao, Lei Sun, Limin Yang, Xingwang Li, Rongmeng Jiang

**Affiliations:** ^1^Beijing Ditan Hospital Capital Medical University, Beijing, China; ^2^Guangdong Provincial Hospital of Traditional Chinese Medicine, Guangdong, China; ^3^Beijing Hospital of Ministry of Health, Beijing, China; ^4^Beijing Chaoyang Hospital Affiliated to Capital Medical University, Beijing, China; ^5^Beijing Haidian Hospital, Beijing, China; ^6^Beijing Luhe Hospital affiliated to Capital Medical University, Beijing, China; ^7^Beijing Sixth Hospital, Beijing, China; ^8^The First Hospital of Shijiazhuang, Shijiazhuang, China; ^9^Xuanwu Hospital, Capital Medical University, Beijing, China; ^10^Hebei Yiling Hospital, Shijiazhuang, China; ^11^China-Japan Friendship Hospital, Beijing, China; ^12^The Third Hospital of Shijiazhuang, Shijiazhuang, China; ^13^National Key Laboratory for Innovation and Transformation of Luobing Theory, Jinan, China

**Keywords:** non-influenza virus pneumonia, Lianhua clear blast particles, Chinese medicine treatment, clinical trials, RCT – randomized controlled trial

## Abstract

**Objective:**

To observe the effectiveness and safety of Lianhua Qingwen granule in the treatment of non-influenza viral pneumonia.

**Methods:**

This study was a multicenter, randomized, double-blind, placebo-controlled trial. Subjects who met the inclusion and exclusion criteria and were clinically diagnosed with viral pneumonia (negative for influenza virus) were randomly divided into the Lianhua Qingwen granule trial group and placebo control group. Patients in the trial group was given Lianhua Qingwen granule, 2 bags at a time, 3 times a day, and the controls were given placebo, with a treatment course of 7 days. Patients’ clinical symptoms and signs, and treatment-associated adverse events were observed. Subjects should be included in the full analysis set (FAS) as long as they were all given the medication and had an effectiveness test performed after randomization. Subjects should be included in the Per Protocol Set (PPS),a subset of the total analysis set, which should contain those with strong compliance, no protocol violations, and complete baseline values for the primary indicators.

**Results:**

A total of 169 subjects were enrolled in 12 subcenters, including 151 (76 in the trial group and 75 in the control group) in the FAS and 140 (68 in the trial group and 72 in the control group) in the PPS. After 7 days of treatment, the clinical symptom relief rates were 82.98% (FAS) and 87.12% (PPS) in the trial group, and 75.11% (FAS) and 76.02% (PPS) in the control group, respectively. The clinical symptom relief rates in the trial group were significantly higher than those in the control group (*p* < 0.001). Significant improvements in single symptoms of cough and expectoration in the trial group were observed compared with the control group (*p* < 0.05). There were no statistical differences in fever, sputum color change, chest pain, muscle pain, dyspnea, chills, and thirst between the two groups (*p* > 0.05).

**Safety:**

There were no significant differences in body weight, vital signs, blood routine, urine routine, stool routine, and blood biochemical indicators (CK, AST, ALT, Cr, and Bun) between the two groups before and after treatment (*p* > 0.05). During treatment, there were no significant differences in the incidence of adverse events and serious adverse events between the two groups (*p* > 0.05).

**Conclusion:**

Lianhua Qingwen granules improved the clinical symptoms of patients with non-influenza virus pneumonia, especially ameliorating cough and expectoration. Lianhua Qingwen granules were associated with good safety.

## Introduction

Pneumonia is a leading cause of death in children and the elderly (1). Among the pathogens that cause community-acquired pneumonia (CAP), the disease burden of viral pneumonia is severely underestimated. In recent years, with the development of molecular biological detection technology, more and more attention has been paid to respiratory virus in CAP (2, 3).

As a common and frequently-occurring disease, the diagnosis and treatment of viral pneumonia are not standard. Influenza virus is the most common respiratory virus, easy to cause lung inflammation, especially influenza A H1N1, H3N2, avian influenza H5N1, H7N9 epidemic, making influenza has become the focus of attention of the medical community, there are more drugs to choose, such as amantadine, rimantadine, oseltamivir, zanamivir, peramivir, etc. There is good evidence of evidence-based medicine for clinical efficacy. There are few studies on the standardized treatment of other respiratory viruses except influenza related viruses. For example, respiratory syncytial virus is also the cause of common viral pneumonia, and for patients with more severe illness, the existing evidence recommends the use of ribazole treatment, and palivizumab/Synagis can also be used. Parainfluenza virus does not have a good treatment drug, some experts recommend the use of ribavirin treatment. The treatment of adenovirus mainly uses cidofovir, *in vitro* data show that cidofovir has a good effect against 14 subtypes of adenovirus, but currently it is mainly used in patients with low immunity, especially in patients with hematopoietic stem cell transplantation. Severe Acute Respiratory Syndromes (SARS) and Middle East Respiratory Syndrome (MERS) are both caused by coronaviruses. Although they account for a small proportion in the whole respiratory tract, they have been the focus of medical attention due to their greater public health hazards. The protease inhibitors lopinavir and ritonavir showed anti-SARS-CoV activity. It can be seen that no matter respiratory syncytial virus, parainfluenza virus, adenovirus or coronavirus, the clinical treatment drugs for pneumonia caused by these non-influenza viruses are mostly empirical drugs, which have a long market time, large side effects, lack of high-level evidence-based medicine support, and clinical efficacy is not accurate. Therefore, it is of practical clinical significance to explore new therapeutic means and develop new drugs. With the development of nucleic acid diagnostic technology, pathogen diagnosis can be well distinguished and clarified clinically.

Traditional Chinese medicine (TCM) has a long history, rich experience and exact clinical efficacy in the treatment of infectious diseases, especially respiratory infectious diseases. With the progress of The Times, the clinical experience of traditional Chinese medicine needs to come up with clinical data in line with modern evaluation standards to prove its efficacy. A standard RCT study was conducted by the National Administration of Traditional Chinese Medicine (4). A total of 147 severe influenza patients were collected, 86% of whom had pneumonia. It is analyzed that the traditional Chinese medicine is heat and toxin obstructing the lung, and the treatment is to clear the heat and detoxize the lung and ventilate the pathogens. Other viruses, such as syncytial virus and adenovirus, cause pneumonia. When there are no other complications in the early stage, the pathogenesis of TCM is mainly heat and poison in the lung. Therefore, the basic pathogenesis of virus associated pneumonia in traditional Chinese medicine is the invasion of warm epidemic virus on the lung, and the lung is closed. When the treatment of Qingwen detoxification, Xuan lung heat.

Lianhua Qingwen granule is a Chinese patent medicine for the treatment of common cold and influenza under the guidance of the theory of collateral disease of traditional Chinese medicine. It has been recommended by many diagnosis and treatment plans and guidelines due to its significant clinical therapeutic effect. The treatment method of Lianhua Qingwen is “clearing away the pestilence, detoxifying the lung heat.” This study evaluated the efficacy and safety of Lianhua Qingwen granule in the treatment of non-influenza viral pneumonia, aiming to provide evidence-based medical evidence to develop standardized treatment strategy for pneumonia caused by viral infection.

## Materials and methods

### Study design

This was a multicenter, randomized, double-blind, placebo-controlled trial to evaluate the clinical efficacy of Lianhua Qingwen Granule in the treatment of non-influenza viral pneumonia.

This study was approved by the Ethics Committee of Beijing Ditan Hospital Capital Medical University, China. (Ethics Approval Number: Jing Di Lun Zi [2015] No. [56] -02). Each subject was informed of the purpose and procedures of the study and the potential benefits and risks of treatment, and signed the informed consent. This study was registered in the Chinese Clinical Trial Registry (ChiCTR-IPR-16007773).

## Subject enrollment

### Inclusion criteria

The inclusion criteria were as follows: (1) Patients who met the diagnostic criteria according to the Chinese Guidelines for Diagnosis and Treatment of Community-acquired Pneumonia issued by the Chinese Thoracic Society (2015 Edition); (2) Clinical diagnosis of viral pneumonia: fever with respiratory symptoms, with or without dyspnea (respiratory rate > 30 times/min); white blood cell (WBC) count was normal or below normal, with or without thrombocytopenia; only those patients undergo chest CT who were based on the doctor’s professional judgment and the doctor’s full understanding of the patient’s condition, fully understand the benefit risk ratio of using CT from the patient’s perspective, and finally obtain the informed consent of the patient before undergoing CT examination, the chest CT examination was consistent with the clinical findings of viral pneumonia; (3) Rapid influenza antigen test result was negative (Shenzhen Miraclean Technology Co., Ltd. Influenza antigen test Real-time RT-PCR. Those with negative results were further screened for respiratory viruses in accordance with the guidelines by the Chinese Center for Disease Control and Prevention (Nanjing Synthgene Medical Technology Co. Ltd. Multiple Respiratory pathogen nucleic acid detection kit PCR-Fluorescent probe method. (4) Patients enrolled within 5 days of disease onset; (5) Patients aged 14 years or older, without gender limitation.

### Exclusion criteria

The exclusion criteria were as follows: (1) Without definitive evidence of bacterial infection (PCT > 1ug/L); (2) patients receiving other antiviral drugs within 1 week; (3) Patients who met the diagnosis criteria of severe pneumonia in accordance with the 2015 Chinese Guidelines for Diagnosis and Treatment of Community-acquired Pneumonia; (4) Chest CT confirmed severe interstitial lung disease, bronchiectasis, and other underlying lung diseases; (5) Patients with positive influenza A/B rapid test results and with streptococcus pneumonia, Legionella pneumonia urine antigen, mycoplasma pneumonia, and positive chlamydia antibody testing; (6) Patients with severe liver and kidney dysfunction: ALT /AST values were 3 times higher than the upper limit of normal value, and blood creatinine was 1.5 times higher than the upper limit of normal value; (7) Patients with previous or current diseases that might affect their participation in the trial and the research results, including malignant, autoimmune, liver and kidney, hematological, neurological endocrine diseases; (8) Patients with diseases such as human immunodeficiency virus infection, hematological disorders, or received treatments such as splenectomy and organ transplantation, which seriously affected their immune system; (9) History of seizures, mental illness, drinking alcohol, and illicit drug abuse; (10) Pregnant or lactating women.

### Discontinuation and withdrawal criteria

The discontinuation and withdrawal criteria were as follows: (1) Subjects who have an acute exacerbation leading to the progression to severe disease during the trial; (2) Subjects who develop allergic reactions or serious adverse events; (3) Subjects who experience serious complications or specific physiological changes during the trial that are unsuitable to continue the study; (4) Subjects with poor medication adherence (<80% of dose) or who no longer receive dosing and testing are considered unsuitable to continue in the trial. The reasons for their withdrawal should be recorded in detail. (5) For whatever reason, the patient is unwilling or unable to continue the trial and requests withdrawal from the trial.

### Usage and detection principle

For those with negative results of the rapid influenza antigen test, the usage is the collected person first rinses his mouth with normal saline, and the sampler puts the swab into sterile normal saline to moisten it (it is forbidden to put the swab into the virus preservation solution to avoid allergies caused by antibiotics), the head of the collected person is slightly tilted, the mouth is wide open, and the “ah” sound is made, exposing the tonsils on both sides, the swab is crossed over the base of the tongue, and the tonsils on both sides of the collected person are wiped back and forth at least 3 times with a little force, and then wiped up and down the posterior pharyngeal wall at least 3 times, and the swab head is immersed in a virus preservation solution containing 2~3ml (isotonic saline solution can also be used, tissue culture solution or phosphate buffer) in the tube, discard the tail and tighten the cap. A throat swab can also be placed in the same tube as a nasopharyngeal swab.Detection principle: The real-time RT-PCR-based method for the detection and identification of influenza viruses includes a series of oligonucleotide primers and dual-labeled Taqman probes for the qualitative identification of influenza viruses in respiratory samples and virus isolated cultures using Real-time RT-PCR assays. Among them, the primers and probes for the detection of influenza A and B viruses are general-purpose detection primers and probes, which can be used for the identification of influenza A and B virus types, respectively. Other primer probes are subtype-specific detection primer probes that can be used to identify seasonal influenza viruses currently circulating in the population as well as avian influenza virus subtypes that can infect humans.

For those rapid influenza antigen test result was negative were further screened for respiratory viruses, the usage is 1. To prepare the reagent, take out the reaction buffer and primer probe Mix in the kit, place it at room temperature, wait for complete thawing, shake and mix, and centrifuge for later use; Take out the detection enzyme solution in the kit, centrifuge and set aside, prepare PCR-Mix according to the number of samples to be prepared N (N = number of samples + 1 tube of positive control + 1 tube of negative control), and divide 20 μL of PCR-Mix per well into the fluorescence quantitative PCR eight-link reaction tube, and immediately put it into cryopreservation below –18°C after the reaction buffer, primer probe Mix and detection enzyme solution are used. 2. Sample processing, after the sample is received, the water bath is used for inactivation at 56°C for 30 min, the collected throat swab sample should be extracted, and the RNA sample should be guaranteed to meet the number of RNA required by the experiment, and the extracted RNA sample should be detected immediately or stored below –70°C (no more than 7 days); At the same time, the corresponding volume of positive and negative controls was extracted. 3. Add 10 μL of each of the negative control extraction RNA, positive control extraction RNA, and RNA to be tested in step 2 to each set reaction tube, close the tube cap tightly, and centrifuge briefly. 4. Conduct an analysis of the results. Detection principle: A number of respiratory pathogen nucleic acid detection kits (PCR-fluorescent probe method) use Taqman fluorescent probe method to design primer probes for fluorescence detection for highly conserved and specific regions such as influenza A virus, influenza B virus, respiratory syncytial virus, adenovirus, rhinovirus and Mycoplasma pneumoniae, etc., using different fluorophores for labeling, the nucleic acids in the detection process are reverse transcribed into cDNA, and in the amplification process, specific primers and probes are bound to the target sequence. The DNA polymerase activity and 5’-3’ exonuclease activity of Taq enzyme were used to achieve complete synchronization between PCR product formation and fluorescence signal accumulation. Qualitative detection of influenza A virus, influenza B virus, respiratory syncytial virus, adenovirus, rhinovirus and Mycoplasma pneumoniae nucleic acid in samples was achieved by detecting different fluorophore signals.

### Randomization and masking

We randomly assigned patients (1,1) to receive treatment with Lianhua Qingwen granule or matching placebo (manufactured by Shijiazhuang Yiling Pharmaceutical Co. Ltd., Shijiazhuang, China) based on the randomization numbers generated with the SAS package (SAS Inc., Cary, United States). The block size was 4 with no stratification. With a competitive recruitment scheme, the sub-site investigators allocated patients in an ascending order. The study medications had an identical color, odor and appearance, except that the placebo did not contain any active ingredient of LHQW. Patients, the study investigators and other staff were masked to treatment allocation until database lock.

## Research methods

Subjects in the experimental group were given Lianhua Qingwen granule (10 bags per box, 6 g per bag), 2 bags at a time, 3 times a day, which was composed of Chinese herbs (Table 1).

**Table 1:** Composition of LHQW.

Botanical name	Family	Used part	Weight
*Forsythia suspensa* (Thunb.) Vahl	Oleaceae	Fructus	255 g
*Lonicera japonica* Thunb.	Caprifoliaceae	Flower bud	255 g
*Gypsum Fibrosum*	–	–	255 g
*Isatis indigotica* Fortune	Brassicaceae	Root	255 g
*Dryopteris crassirhizoma* Nakai	Dryopteridaceae	Rhizoma	255 g
*Houttuynia cordata* Thunb.	Saururaceae	Whole plant	255 g
*Ephedra sinica* Stapf	Ephedraceae	Stem	85 g
*Glycyrrhiza uralensis* Fisch.	Leguminosae	Rhizoma	85 g
*Pogostemon cablin* (Blanco) Benth.	Labiatae	Whole plant	85 g
*Armeniaca sibirica* (L.) Lam.	Rosaceae	Seed	85 g
*Rhodiola crenulata*	Crassulaceae	Rhizoma	85 g
*Rheum palmatum* L.	Polygonaceae	Rhizoma	51 g
*Mentha haplocalyx* Briq.	Mentha	Aerial part	7.5 g

The control group subjects were given placebo, 2 bags at a time, 3 times a day, and the treatment course was 7 days. The placebo of Lianhua Qingwen granules was made of dextrin (59.45%), lactose (39.63%), caramel (39.63%), sunset yellow (0.01%), tartrazine (0.02%), and menthol (0.14%), which did not contain inert substances. All test drugs were provided by Yiling Pharmaceutical. Clinical symptoms and signs were followed up every day for 7 days. Blood samples were collected for routine biochemical tests on the 8th day. Combined administration and adverse events were recorded.

In addition to research drugs, basic conventional treatment drugs refer to the Guidelines for Diagnosis and Treatment of Community-acquired Pneumonia issued by the Chinese Thoracic Society in 2015. Antiviral drugs and Chinese medicines with antiviral effects, such as Xiyanping, Reduning, Tanreqing, Jinhuaqinggan, and Banlangen, were not allowed to be used within one week before participating in this study and after entering the randomization period. The name, dosage, frequency, and treatment time of antibiotics, hormones, antipyretic analgesics, and cough and asthma relief drugs that were used should be recorded in the case report form. Taking medication for other diseases must also be recorded in the case report form. Subjects could take medications to control hypertension, angina, and diabetes.

### Assessment

#### Efficacy assessment

The main end point of efficacy was the decrease of clinical symptom score at day 7, which was the sum of eight symptom scores including fever, cough, phlegm, chest pain, muscle pain, chills, dyspnea, and thirst. Secondary efficacy endpoints included the proportion of patients whose blood oxygen saturation returned to normal; the relief rate of individual symptoms such as fever, cough and expectoration (symptom relief was defined as: when the pre-treatment symptom score was >2 points, less than 2 points was considered symptom relief; when the pre-treatment symptom score was ≤2 points, 0 point was considered symptom relief); length of hospital stay; RICU transfer time; incidence of complications; and antibiotic utilization rate.

According to the specific conditions of the patients, the study doctors decide whether to be hospitalized or treated at home. The patients receiving treatment at home will visit the hospital and complete the relevant efficacy evaluation, laboratory and imaging examinations according to the visit time stipulated in the program. Participants receiving treatment at home filled out a symptom score and medication status on a daily basis, and the investigators assessed the subjects’ medication compliance based on the diary cards filled out by the patients and the amount of medication withdrawn.

#### Safety assessment

Safety endpoints comprised the incidence of adverse events; clinical laboratory indicators including blood routine, urine routine, serum biochemistry examination (CK, ALT, AST, Bun, Cr); 12-lead ECG examination; physical examination: a. complete physical examination: general condition (including height and weight), vital signs (including blood pressure and pulse rate), skin (including hair and nails), EENT, neck/thyroid, chest/lung, cardiovascular system, abdominal/gastrointestinal system, genitourinary system, nervous system, lymph, and skeletal muscle. b. simplified physical examination: general information (including weight), vital signs (including blood pressure and pulse rate), chest/lung, cardiovascular system. c. vital signs: sitting blood pressure, pulse rate, respiratory rate, and heart rate.

### Statistical analysis

#### Sample size estimation

The primary endpoint is the reduction in clinical symptom score. The authors assumed that the efficacy rate of the trial group was 80% and that of the control group was 60% according to the clinical experience. The patients in the trial group and the control group were allocated in the ratio of 1:1 (a = 0.05 [bilateral] and power = 0.80) and the total sample size of this study was finally set to 164, including 82 cases in the trial group and 82 cases in the control group according to Power Analysis and Sample Size (PASS)15.0 calculation.

#### Clinical data analyses and outputs

Statistical analysis was performed using SAS 9.3 software. *p* value indicates a statistical difference, *p* < 0.05 indicates a statistically significant difference. Two-sided tests was used for baseline comparison before treatment between the two groups. Group *T*-test or Wilcoxon rank-sum test was used to compare quantitative data. Chi-square test or exact probability method was used to compare categorical data. Wilcoxon rank-sum test was used to compare grade data.

A covariance model was constructed with clinical symptom remission rate as the dependent variable, and clinical symptom scores, grouping, and baseline characteristics as independent variables. The least-square mean and 95% confidence interval were calculated and compared between the trial and control groups. The treatment in the trial group achieved higher efficacy compared with the control group when H0 was rejected at the significance level of α = 0.05, or the lower limit of 95% CI was greater than 0 when comparing the difference in clinical symptom remission rate between the two groups.

## Results

### Study recruitment and follow-up

From January 2016 to December 2018, a total of 169 subjects meeting the study requirements were enrolled in 12 hospitals.

**Figure 1:**
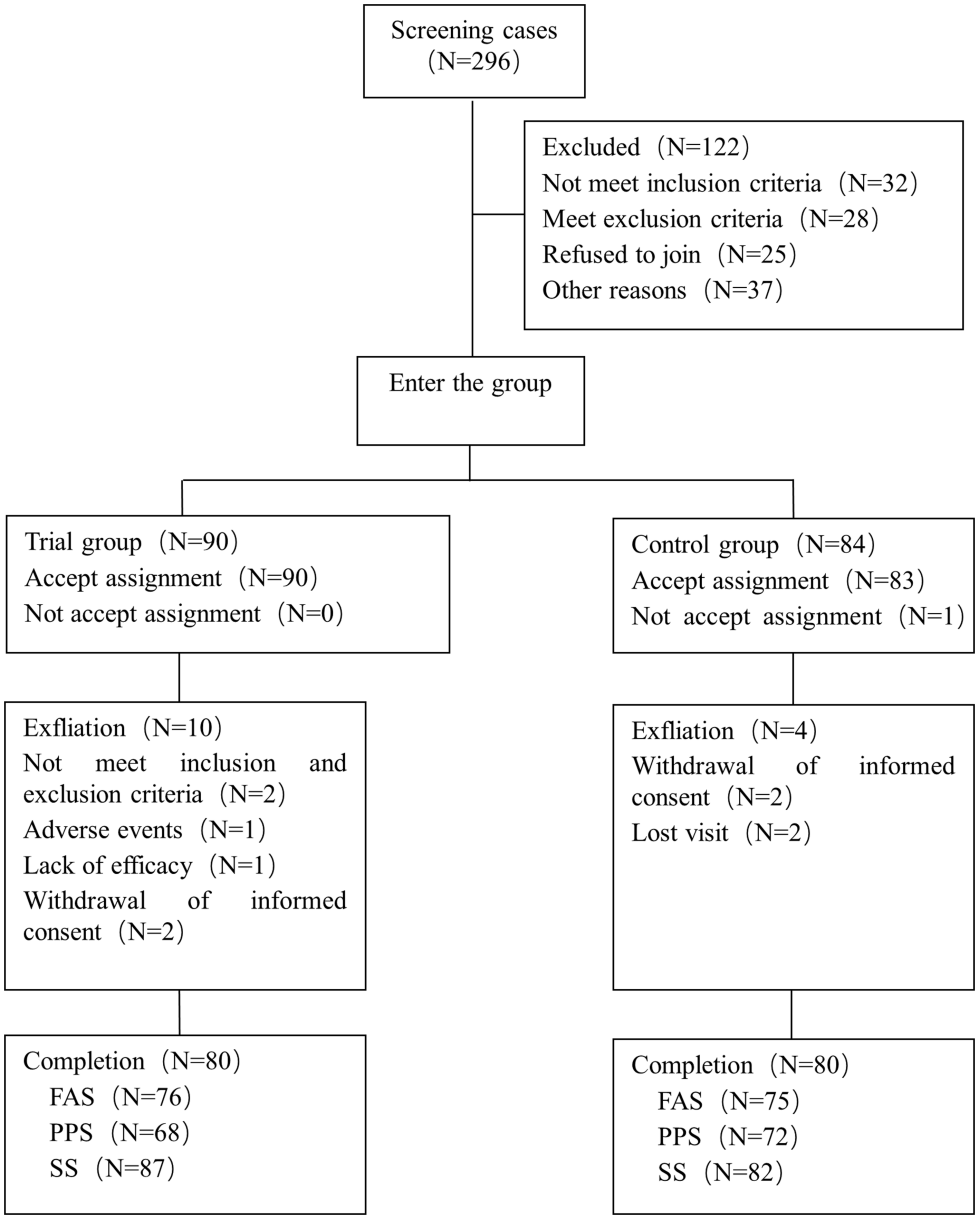
Study flow diagram.

A total of 296 subjects were screened and 174 subjects were randomly enrolled, including 90 in the trial group and 84 in the control group. There were 151 subjects, including 76 in the trial group and 75 in the control group, in the FAS set. PPS set comprised 140 subjects, including 68 in the trial group, and 72 in the control group. SS set covered 169 subjects, including 87 in the trial group and 82 in the control group. A total of 160 subjects completed the study, including 80 in the trial group and 80 in the control group. Totally, 14 subjects dropped out the trial, including 10 in the trial group, and 4 in the control group (Figure 1).

### Subject characteristics

According to FAS set data analysis, the average age of the subjects was 46.83 ± 20.94 years in the trial group, and 43.29 ± 19.72 years in the control group. Subjects in the trial group had a body weight of 63.74 ± 14.55 Kg and a BMI of 22.98 ± 4.17 Kg/m^2^, and those in the control group had a body weight of 64.05 ± 13.27 Kg and a BMI of 23.43 ± 3.86 Kg/m^2^. The average body temperature of the subjects in the trial and control group was 37.71 ± 1.09 and 37.83 ± 1.09, respectively. There were no significant differences in vital signs (body temperature, respiration and heart rate, pulse, and blood pressure), symptoms and signs of runny nose, sore throat, rales, headache and fatigue, and diarrhea during the screening period between the two groups (*p* > 0.05). There was no significant difference in clinical symptom scores (the sum scores of eight symptoms including fever, cough, phlegm, chest pain, muscle pain, chills, dyspnea, and thirst) between the two groups during the screening period (*p* > 0.05). Patients in both groups were diagnosed with non-influenza viral pneumonia. The baseline data of the two groups were comparable (*p* > 0.05) (Table 2).

**Table 2:** Baseline demographic and clinical characteristics of the full-analysis set.

	LHQW group (*N* = 76)	Placebo group (*N* = 75)	Total (*N* = 151)
Age (years), Mean ± SD	46.83 ± 20.94	43.29 ± 19.72	45.07 ± 20.35
Females, n(%)	42 (55.26)	46 (61.33)	88 (58.28)
males, n(%)	34 (44.74)	29 (38.67)	63 (41.72)
Height (cm), Mean ± SD	166.01 ± 7.90	164.93 ± 7.86	165.48 ± 7.87
BMI (kg/m^2^), Mean ± SD	22.98 ± 4.17	23.43 ± 3.86	23.20 ± 4.01
Vital signs			
Body temperature (°C), Mean ± SD	37.71 ± 1.09	37.83 ± 1.09	37.77 ± 1.09
Breath (Times/min), Mean ± SD	19.66 ± 2.16	19.84 ± 2.27	19.75 ± 2.21
Heart rate (Times/min), Mean ± SD	87.17 ± 12.06	90.92 ± 14.28	89.03 ± 13.30
Pulse (Times/min), Mean ± SD	87.17 ± 12.06	90.88 ± 14.30	89.01 ± 13.31
Systolic blood pressure (mmHg), Mean ± SD	119.79 ± 14.80	120.99 ± 15.92	120.38 ± 15.33
Diastolic blood pressure (mmHg), Mean ± SD	73.25 ± 9.82	74.09 ± 8.96	73.67 ± 9.38
Symptoms or signs			
Running nose, yes, n (%)	12 (15.79)	11 (14.67)	23 (15.23)
Running nose, no, n (%)	64 (84.21)	64 (85.33)	128 (84.77)
Angina, yes, n (%)	28 (36.84)	25 (33.33)	53 (35.10)
Angina, no, n (%)	48 (63.16)	50 (66.67)	98 (64.90)
Nasal obstruction, yes, n (%)	9 (11.84)	15 (20.00)	24 (15.89)
Nasal obstruction, no, n (%)	67 (88.16)	60 (80.00)	127 (84.11)
Rale, yes, n (%)	25 (32.89)	27 (36.00)	52 (34.44)
rale, no, n (%)	51 (67.11)	48 (64.00)	99 (65.56)
Headache and fatigue, yes, n (%)	39 (51.32)	38 (50.67)	77 (50.99)
Headache and fatigue, no, n (%)	37 (48.68)	37 (49.33)	74 (49.01)
diarrhea, yes, n (%)	3 (3.95)	7 (9.33)	10 (6.62)
diarrhea, no, n (%)	73 (96.05)	68 (90.67)	141 (93.38)
Self-medication before visit, yes, n (%)	43 (56.58)	46 (61.33)	89 (58.94)
Self-medication before visit, no, n (%)	33 (43.42)	29 (38.67)	62 (41.06)

### Clinical outcomes

After 7 days of treatment, the clinical symptom relief rates of the trial groups were 82.98% (FAS) and 87.12% (PPS), respectively, and the clinical symptom relief rates of the control group were 75.11% (FAS) and 76.02% (PPS), respectively. The trial group achieved a better efficacy compared with the control group (*p* < 0.001). The difference in remission rate between the two groups was 7.87 [95%CI (0.42, 15.32)] (FAS) and 11.10 [95%CI (4.61, 17.58)] (PPS), respectively. ANCOVA showed that the lower limit of 95%CI was greater than 0 when comparing the difference in remission rate of clinical symptoms between the two groups, indicating that the treatment in the trial group was superior to the control group. The results of FAS and PPS analyses were consistent (Tables 3–5).

**Table 3:** Comparison of clinical symptom relief rate between the two groups after 7 days of treatment.

		FAS		PPS	
Indicator		Trial group	Control group	Trial group	Control group
Clinical symptom remission rate (%)	Mean (SD)	82.98 (24.00)	75.11 (22.28)	87.12 (18.01)	76.02 (20.62)
	P*	0.004		<0.001	

Clinical symptom remission rate (%) = (clinical symptom score before treatment – clinical symptom score after treatment)/score before treatment X100. *Wilcoxon rank sum test.

**Table 4:** Optimal test of 7-day clinical symptom relief rate between the two groups.

		FAS		PPS	
Indicator	Control	Remission rate	95%CI	Remission rate	95%CI
Clinical symptom remission rate (%)	control group	7.87	0.42,15.32	11.10	4.61,17.58

The optimal threshold is 0.00%.

**Table 5:** The corrected mean difference in 7-day clinical symptom remission rate between the two groups.

	FAS		PPS	
	Trial group	Control group	Trial group	Control group
Fixed mean	85.53	77.48	88.39	77.01
Standard error	2.98	2.95	2.59	2.48
Test statistic t	2.116		3.467	
P	0.036		<0.001	
Corrected mean difference	8.05		11.38	
95%CI	0.53,15.57		4.88,17.88	

Baseline clinical symptom score before treatment was a covariate, and there was no interaction between the centers and the subgroups.

On the 7th day of treatment, subjects in the trial group had significant improvements in cough and expectoration compared with those in the control group (*p* < 0.05). There were no statistical differences in fever, sputum color change, chest pain, muscle pain, dyspnea, chills, thirst, and other symptoms (*p* > 0.05) (Tables 3–6).

**Table 6:** Comparison of single symptom relief between the two groups.

Symptoms	FAS			PPS		
	Trial group	Control group	*p*-value	Trial group	Control group	*p*-value
Fever	68/69 (98.55%)	71/72 (98.61%)	0.949	66/67 (98.51%)	70/71 (98.59%)	0.949
Cough	64/69 (92.75%)	58/72 (80.56%)	0.018	62/67 (92.54%)	57/71 (80.28%)	0.019
Expectoration	52/69 (75.36%)	41/72 (56.94%)	0.019	51/67 (76.12%)	40/71 (56.34%)	0.010
Chest pain	69/69 (100.00%)	70/72 (97.22%)	0.144	67/67 (100.00%)	69/71 (97.18%)	0.144
Muscle soreness	69/69 (100.00%)	70/72 (97.22%)	0.151	67/67 (100.00%)	69/71 (97.18%)	0.151
Chills	68/69 (98.55%)	71/72 (98.61%)	0.942	66/67 (98.51%)	70/71 (98.59%)	0.942
Difficulty breathing	69/69 (100.00%)	69/72 (95.83%)	0.070	67/67 (100.00%)	68/71 (95.77%)	0.057
Thirst	69/69 (100.00%)	71/72 (98.61%)	0.317	67/67 (100.00%)	70/71 (98.59%)	0.317

Some subjects in FAS were dropout (exclusion) and were not included in the PPS, of which 1 patient withdrew informed consent, 3 patients had poor compliance, 1 patient had adverse events, 1 patient lacked efficacy withdrawal, and 5 patients did not come to the hospital on time for a revisit visit (Table 7).

**Table 7:** List of dropout/exclusion subjects.

						Analysis dataset		
No	Group	Enrollment time	Dropout/Exclusion	Date of last visit	Cause of dropout/exclusion	PPS	FAS	SS
1	Trial group	2016-12-13	Dropout	2016-12-16	Withdrew informed consent	No	Yes	Yes
2	Trial group	2018-02-24	Exclusion	2018-03-05	Poor compliance	No	Yes	Yes
3	Trial group	2017-01-16	Dropout	2017-01-17	The patient did not come to the hospital on time for a revisit visit	No	Yes	Yes
4	Trial group	2018-01-26	Dropout	2018-01-30	Adverse events	No	Yes	Yes
5	Trial group	2017-09-08	Exclusion	2017-09-12	Poor compliance	No	Yes	Yes
6	Control group	2018-05-01	Exclusion	2018-05-08	Poor compliance	No	Yes	Yes
7	Trial group	2017-11-11-	Dropout	2017-11-14	The patient did not come to the hospital on time for a revisit visit	No	Yes	Yes
8	Trial group	2017-07-26	Dropout	2017-07-30	Lack of efficacy and withdrawal	No	Yes	Yes
9	Control group	2017-11-12	Dropout	2017-11-19	The patient did not come to the hospital on time for a revisit visit	No	Yes	Yes
10	Trial group	2017-12-12	Dropout	2017-12-15	The patient did not come to the hospital on time for a revisit visit	No	Yes	Yes
11	Control group	2017-12-28	Dropout	2017-12-31	The patient did not come to the hospital on time for a revisit visit	No	Yes	Yes

### Safety

There were no significant differences in body weight, vital signs, blood routine, urine routine, stool routine, and blood biochemical results (CK, AST, ALT, Bun, and Cr) between the two groups before and after 7 days of treatment (*p* > 0.05). During treatment, there was no significant difference in the incidence of adverse events and serious adverse events between the two groups (*p* > 0.05) (Table 8).

**Table 8:** The incidence of adverse events between the two groups.

	Trial group			Control group			
	Times	Number of people	Percentage	Times	Number of people	Percentage	*p*-value
Adverse events	30	23	26.44%	51	30	36.59%	0.185
Adverse reactions	4	4	4.60%	3	2	2.44%	0.683
Severe adverse events	0	0	0.00%	0	0	0.00%	–
Severe adverse reaction	0	0	0.00%	0	0	0.00%	–
Adverse event leading to abscission	1	1	1.15%	0	0	0.00%	1.000

Adverse reactions refer that the relationship with the study drug is positive, possible, and cannot be determined. Serious adverse events refer that occur at any dose of the investigational drug or at any time during the observation period. Serious adverse reactions refer that cause death, cancer, teratogenesis, birth defects, life-threatening or permanent or significant damage to the human body or organ.

## Discussion

Pneumonia is estimated to cause the death of more than 3 million people worldwide each year. In the last decade, with the improvements in sensitivity, availability, and affordability of molecular pathogen detection methods, there has been a new understanding of the structure of pneumonia (3). As one of the pathogens causing community-acquired pneumonia, pneumonia virus has attracted more and more attention. At least 20 viruses, such as influenza A, B, and C viruses, respiratory syncytial viruses, rhinoviruses, parainfluenza viruses, adenoviruses, human metapneumoviruses, human boca viruses, and coronaviruses, have been found to cause pneumonia. The normal lower respiratory tract is not colonized by viruses. Viral pneumonia is mainly caused by virus infection, usually affecting the upper respiratory tract down to the respiratory tract. Viral pneumonia is often highly seasonal and contagious, with a high incidence in winter and spring, and is easy to spread. Some special populations, such as the elderly, children and those with chronic underlying diseases, are susceptible to severe pneumonia triggered by bacterial or fungal infection, resulting in multiple organ dysfunction (7, 8).

For the treatment of pathogen-induced pneumonia, currently, only antiviral drugs against influenza virus have been proved to be effective. No definitive effects of other antiviral drugs for treating viral pneumonia have been reported. Traditional Chinese Medicine has a rich historical record in the treatment of viral infectious respiratory diseases and has the advantages of a short course of disease, less relapse after antipyretic, and quick elimination of systemic symptoms.

Lianhua Qingwen granule is a pure Chinese medicine preparation, and its components include lianqiao, jinyinhua, zhimahuang, chaokuxingren, shigao, banlangen, mianmaguanzhong, yuxingcao, guanghuoxiang, dahuang, hongjingtian, bohenao, and gancao. Jinyinhua and lianqiao have the effect of clearing away heat, relieving the toxin, and further dispelling wind and heat from the body. Banlangen, guanzhong, and yuxingcao can also clear heat and detoxify, and are important antiviral drugs in Traditional Chinese Medicine. Lianhua Qingwen granules are effective in treating influenza virus, and also have an antiviral effect on common respiratory viruses. At the same time, they have antibacterial, antipyretic, analgesic, anti-inflammatory functions, thereby relieving cough, reducing phlegm, and adjusting immune responses (2, 9, 10).

Some possible shortcomings of this trial are that patients were treated with antibiotics, which are not recommended in any current guidelines for uncomplicated viral pneumonia. About viral pneumonia, it is correct that various guidelines do not recommend the use of antibiotics. The use of antibiotics in viral pneumonia is mainly based on doctors’ experience and the habit of fearing secondary bacterial infections after viral infections (11). This is especially common in developing countries, including China (12). Regardless of whether it is pneumonia caused by influenza viruses (13) or pneumonia caused by covid-19 (14) infection, the misuse of antibiotics still exists. There has even been a situation of antibiotic overuse, which is a public health issue that we need to address. In this study, considering the practical situation in clinical settings, there was no specific agreement on the types of antibiotics used. The choice of antibiotics was mainly based on physicians’ prescribing habits and the availability of antibiotics. The antibiotics primarily used in this project include Moxifloxacin, Piperacillin Tazobactam, Levofloxacin, Minocycline, Ceftriaxone, Azithromycin, Ceftazidime, and others. There were some differences in the antibiotics used by different research centers, but there was no statistically significant difference in the use of antibiotic types between the two groups, and it did not have a specific impact on the evaluation of the efficacy of Lianhua Qingwen Capsules. Of course, in future research, efforts should be made to further strengthen the management of antibiotic use.

Our results showed that after 7 days of treatment, for both FAS and PPS sets, the remission rate of clinical symptoms and the total score of clinical symptoms in the trial group were improved compared with the control group, with statistical significance. Moreover, there were no significant differences in safety evaluation indexes, such as body weight, vital signs, blood routine, urine routine, stool routine, creatine kinase, alanine aminotransferase, aspartate aminotransferase, creatinine and urea nitrogen, between the two groups. There were no significant differences in the incidence of adverse events and serious adverse events between the two groups, suggesting that Lianhua Qingwen granules were well tolerated and safe.

## Conclusion

Lianhua Qingwen granule treatment improved the clinical symptoms of patients with non-influenza virus pneumonia, especially the symptoms of cough and expectoration, and was associated with good safety.

## Data availability statement

The original contributions presented in the study are included in the article/supplementary material, further inquiries can be directed to the corresponding authors.

## Ethics statement

The studies involving humans were approved by Ethics Committee of Beijing Ditan Hospital Capital Medical University, China. The studies were conducted in accordance with the local legislation and institutional requirements. The participants provided their written informed consent to participate in this study.

## Author contributions

CM: Writing – original draft, Writing – review & editing. BC: Writing – original draft, Writing – review & editing. YL: Writing – original draft, Writing – review & editing. LG: Writing – original draft, Writing – review & editing. JD: Project administration, Writing – original draft. ZX: Project administration, Writing – original draft. LW: Project administration, Writing – original draft. ZH: Project administration, Writing – original draft. XN: Project administration, Writing – original draft. SF: Project administration, Writing – original draft. BC: Project administration, Writing – original draft. LS: Project administration, Writing – original draft. LY: Project administration, Writing – original draft. XL: Conceptualization, Supervision, Writing – original draft. RJ: Conceptualization, Supervision, Writing – original draft.
